# Fouling and Chemical Cleaning Strategies for Submerged Ultrafiltration Membrane: Synchronized Bench-Scale, Full-Scale, and Engineering Tests

**DOI:** 10.3390/membranes14120251

**Published:** 2024-11-26

**Authors:** Xiwang Zhu, Chengyue Fan, Yichen Fang, Wenqing Yu, Yawei Xie, Hongyuan Liu

**Affiliations:** 1 College of Civil Engineering, Zhejiang University of Technology, Hangzhou 310023, China; 211122060055@zjut.edu.cn (X.Z.); 1112006010@zjut.edu.cn (C.F.); 221122060107@zjut.edu.cn (Y.F.); 2 Zhejiang Supcon Information Co., Ltd., Hangzhou 310056, China; yuwenqing@supconit.com

**Keywords:** submerged ultrafiltration membrane, flux recovery, membrane fouling, chemical cleaning, fouling control strategies

## Abstract

This study investigated membrane fouling issues associated with the operation of a submerged ultrafiltration membrane in a drinking water treatment plant (DWTP) and optimized the associated chemical cleaning strategies. By analyzing the surface components of the membrane foulant and the compositions of the membrane cleaning solution, the primary causes of membrane fouling were identified. Membrane fouling control strategies suitable for the DWTP were evaluated through chemical cleaning tests conducted for bench-scale, full-scale, and engineering cases. The results show that the membrane foulants were primarily composed of a mixture of inorganics and organics; the inorganics were mainly composed of Al and Si, while the organics were primarily humic acid (HA). Sodium citrate proved to be the most effective cleaning agent for inorganic fouling, which was mainly composed of Al, whereas sodium hypochlorite (NaClO) combined with sodium hydroxide (NaOH) showed the best removal efficiency for organic fouling, which predominantly consisted of HA and Si. However, sodium hypochlorite (NaClO) combined with sodium hydroxide (NaOH) showed the best removal efficiency for organic fouling and Si; organic fouling predominantly consisted of HA. Based on the bench-scale test results, flux recovery was verified in the full-scale system. Under a constant pressure of 30 kPa, the combined acid–alkali cleaning achieved the best flux recovery, restoring the flux from 22.8 L/(m^2^·h) to 66.75 L/(m^2^·h). In the engineering tests, combined acid–alkali cleaning yielded results consistent with those of the full-scale tests. In the practical engineering cleaning process, adopting a cleaning strategy of alkaline (NaClO + NaOH) cleaning followed by acidic (sodium citrate) cleaning can effectively solve the membrane fouling problem.

## 1. Introduction

In recent years, the ultrafiltration (UF) membrane process has seen significant advancements, leading to its increasing application in water treatment processes [1,2]. Drinking water treatment plants (DWTPs) have adopted the UF membrane process due to its high water yield, modularity, and efficient, clean operation. Additionally, the UF process demonstrates strong adaptability to varying raw water conditions [3,4,5]. Compared to conventional water treatment methods, the membrane technique offers several advantages, including a shorter treatment process, lower energy consumption, reduced footprint, and superior removal efficiency for colloids, organics, and pathogenic microorganisms such as Giardia and Cryptosporidium [6]. Nevertheless, membrane fouling remains a significant challenge in practical applications, hindering the further development of the UF membrane technique [7].

Membrane fouling can be categorized into inorganic fouling, natural organic fouling, and biological fouling [8,9]. Inorganic fouling primarily consists of calcium sulfate, calcium carbonate, calcium phosphate, metal oxides, hydroxides (especially Si and Al) [10,11,12], colloidal substances, and other inorganic particulates. These substances interact with and deposit on the membrane surface and within its pores, with dissolved metals forming an oxidation layer on the membrane. Numerous pieces of research indicate that fouling caused by colloidal particulates in river water is mostly reversible [13,14]. The impact of inorganics on the membrane is relatively well understood; they form a filter cake layer on the membrane surface, increasing resistance to water flow, which generally results in reversible fouling.

Natural organic fouling, on the other hand, comprises a variety of organic molecules, forming a more complex fouling system [15]. Yue [16] found that humic acid (HA), proteins, and polysaccharides are the primary macromolecular organics contributing to severe membrane fouling, with micromolecular organics having a lesser impact. Unstable proteins can denature due to changes in temperature and hydraulic conditions [17] and tend to adsorb rapidly onto the membrane surface [18]. HA adsorption or deposition leads to pore narrowing or clogging, resulting in a decrease in membrane flux and causing irreversible fouling [19]. Colloidal depositions involving proteins, polysaccharides, and other colloidal substances form a denser filter cake layer on the membrane surface.

Biological fouling occurs when microorganisms proliferate on the membrane surface, producing a significant amount of extracellular polymers. This process tightens the cake under the influence of inlet pressure, thereby intensifying membrane fouling [20]. The diverse foulant types present in natural water bodies contribute to complex fouling mechanisms and synergistic effects. For example, the presence of various inorganic ions can exacerbate HA fouling in UF membranes [21]. When HA coexists with inorganic ions, these ions influence the extent and mechanism of HA fouling through charge shielding, charge neutralization, complexation, and bridging effects [22].

Chemical cleaning and physical cleaning are two main processes mitigating membrane fouling. Acidic cleaning reagents for inorganic minerals, metal oxides, and calcium and magnesium carbonates have a good cleaning effect; however, strong acids can significantly alter the pH of the solution. Too low a pH can damage the integrity of the membrane [23]. Sodium citrate is a weak acid and chelating reagent that can easily form chelates and complexes with metal ions; it also significantly enhances the cleaning efficiency [24]. Alkaline detergent is usually used to clean membrane fouling from organics and microorganisms. Sodium hydroxide (NaOH) is one alkaline detergent commonly used to clean membrane foulants, mainly to enhance the solubility of the foulant in water, but it lacks a certain buffering capacity [25]. In the removal of organics, NaClO has a better cleaning effect than NaOH [26,27], which is attributed to the fact that NaClO may cause membrane swelling, which helps to flush out material that may be trapped in the pores [28]. There exist different complex interactions between different foulants and membranes, and different chemical cleaning reagents have specific adaptive ranges [29]. Currently, membrane cleaning methods and strategies do not adequately address the complexities of membrane fouling, such as the diversity of foulants and fouling behaviors. There is a notable lack of systematic research on optimizing membrane cleaning strategies, particularly in terms of bench-scale tests, full-scale tests, and engineering applications. To develop an effective cleaning program, it is crucial to accurately identify membrane foulants and apply targeted fouling control measures based on the specific type of fouling encountered. Thus, optimizing membrane cleaning requires a thorough understanding of the foulants involved, enabling the development of tailored strategies to effectively manage membrane fouling.

The UF membranes used in this work were taken from the submerged ultrafiltration membrane (SUM) of the drinking water treatment plant (DWTP). After identifying the membrane-fouling substances, an appropriate cleaning control strategy was implemented to effectively manage membrane fouling. The fouled membrane was chemically cleaned in the laboratory using sodium citrate, sodium hypochlorite (NaClO), and a combination of NaClO + NaOH. The fouling condition was assessed by analyzing the eluates’ organic chemical indicators, materialistic components, and metal ion concentrations, and the membrane’s surface characteristics. Based on this analysis, suitable cleaning reagents and operating conditions were selected. Membrane flux recovery was evaluated through full-scale tests, and transmembrane pressure (TMP) recovery was assessed via engineering tests. This study explored effective chemicals and cleaning methods, offering practical recommendations for the DWTP to address membrane fouling issues.

## 2. Materials and Methods

### 2.1. Characteristics of DWTP

**Figure 1:**
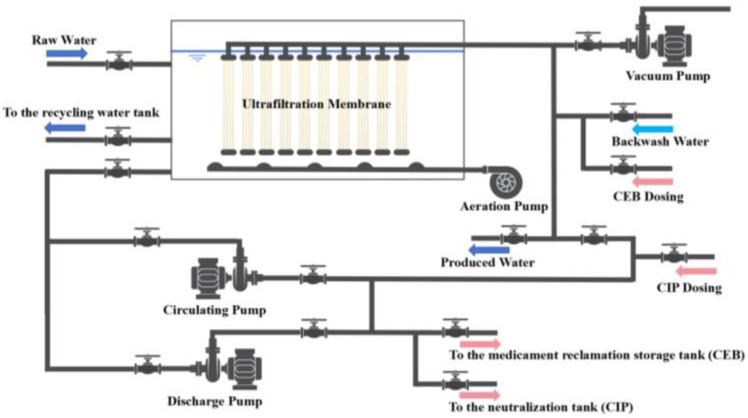
Process flow diagram of ultrafiltration system in DWTP.

The DWTP, located in a city in eastern Zhejiang Province, has a water supply capacity of 500,000 m^3^/d. Raw water is sourced from a local reservoir, and the water purification process includes folded plate flocculation sedimentation, UF, and disinfection. A schematic of the DWTP’s ultrafiltration system is shown in Figure 1. The system utilizes PVDF hollow-fiber UF membranes (Tianjin Motimo Membrane Technology, Tianjin, China) with a total of 40 membrane groups and a retention pore size of 30 nm. Each group is split into two membrane units, with each unit containing 26 ultrafiltration membrane modules. Every membrane module offers an effective filtration area of 35 m^2^, and the UF system operates using a constant flow filtration method.

The UF system performs a 90 s backwash every hour, with a hydraulic backwash intensity of 38 L/(m^2^·h) and an aeration intensity of 142 m^3^/(m^2^·h). Initially, surface foulants on the membrane filaments are removed through hydraulic backwashing. However, as the operating time increases, it becomes challenging to restore TMP through physical cleaning alone, necessitating periodic chemical cleaning. Chemical reagents are introduced into the membrane tank via a dosing pump to circulate through the system for the soaking and restorative cleaning of the membrane components. Once TMP is restored, the reagents are discharged, and production resumes after thoroughly rinsing away any residual chemicals.

Membrane foulants were analyzed in bench-scale tests, membrane flux recovery was evaluated in full-scale tests, and TMP recovery was assessed in engineering tests.

The seasonal raw water temperature, turbidity, COD_Mn_, and other indicators of this DWPT change significantly with the season (Table 1). Furthermore, the quality of raw water in summer is inferior to that in the other seasons. Therefore, the full-scale and engineering tests in this paper were carried out in the summer to deal with the worst conditions.

### 2.2. Membrane Foulant Identification and Cleaning Experiment

The key substances in the membrane foulants were identified by analyzing the physical and chemical properties of the membrane eluates, combined with a component analysis of the fouled membranes. Full-scale tests were then conducted to verify the membrane flux recovery achieved with each of the cleaning methods. The reagents and combinations used in the cleaning experiments were selected to fit the actual engineering scenario, based on the existing cleaning agents and cleaning combinations used in the DWTP. The effect of NaOH was not examined separately. Previous studies reported that NaClO has a better cleaning effect than NaOH, and the combined NaClO + NaOH has more cleaning efficiency [26,27].

(1) Bench-scale tests

Alkaline washing was employed to remove organics, acid washing was used to eliminate inorganic metal ions, scaling, and oxides, and ultrasonic treatment was applied to address reversible fouling. This test analyzed the drainage from these cleaning methods to determine the nature of the membrane foulants. The effectiveness of citric acid in removing organics and alkali in eliminating inorganics was not specifically examined. Chemical solutions of varying concentrations were prepared: sodium citrate (1000 mg/L, 2000 mg/L, 3000 mg/L), NaClO (500 mg/L, 1000 mg/L, 1500 mg/L), and NaOH (250 mg/L). Membrane filament samples were soaked in different chemical cleaning reagents for 24 h, after which the composition of the soaking solutions was analyzed. Ultrapure water was used to rinse the soaking solution from the surface of the membrane filaments. After cleaning, the membrane filaments were freeze-dried, and their surface characteristics were further analyzed.

(2) Full-scale tests

**Figure 2:**
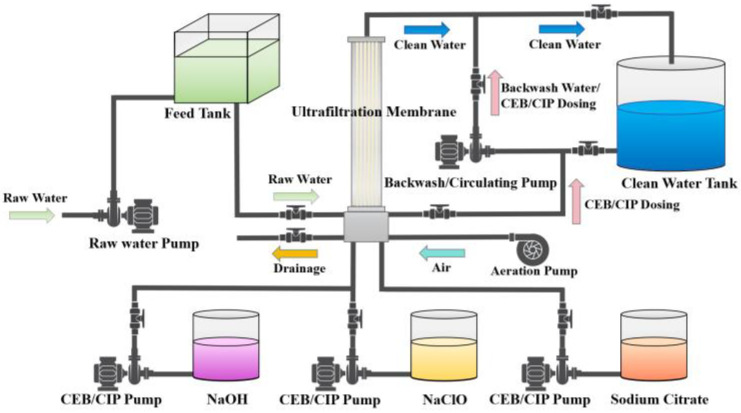
Schematic diagram of full-scale membrane system.

The bench-scale tests focused solely on analyzing the eluent and the changes in the membrane surface post-cleaning without evaluating the membrane flux recovery. Since membrane flux recovery is the most direct and crucial parameter for assessing cleaning effectiveness, full-scale tests were conducted to evaluate the impact of chemical reagents on fouled membrane cleaning. The UF unit was in a DWTP where surface water was used as the raw water in Ningbo, China, and the feed water of the UF was taken from the sand-filter-produced water. The total membrane filtration area of 35 m^2^ with a designed flux is 1 m^3^/h. The filtration process operates for 120 min per cycle, as shown in Figure 2. The cleaning process begins with a 60 s aeration, followed by an air–water backwash at an intensity of 30 L/(m^2^·h), with the membrane tank being emptied every five cycles.

The effectiveness of the three membrane cleaning methods was evaluated: (a) sodium citrate cleaning, (b) NaClO cleaning, and (c) NaClO combined with NaOH cleaning. The concentrations of the above chemicals were determined based on bench-scale tests.

### 2.3. Analytical Methods and Chemicals

#### 2.3.1. Chemicals and Materials

NaOH (AR-grade) and sodium citrate (99.5–100%) were purchased from Shanghai McLean Biochemical Technology Co, Shanghai, China. NaClO (AR-grade, available chlorine 5%) was purchased from Sinopharm Chemical Reagent Co, Shanghai, China.

The membranes used in the tests were PVDF hollow-fiber UF membranes (Tianjin Motimo Membrane Technology, Tianjin, China), which were produced via the thermally induced phase separation (TIPS) process. The characteristics of the membranes are shown in Table 2.

**Figure 3:**
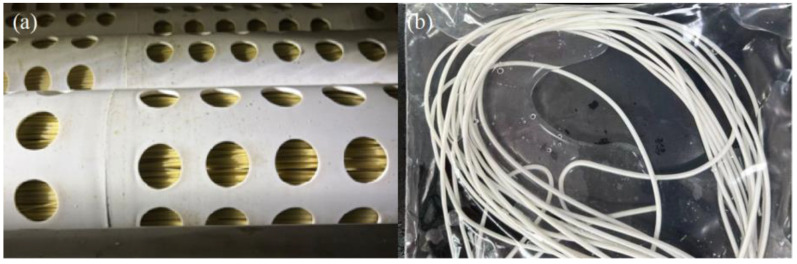
The membrane filaments: (**a**) fouled, (**b**) virgin.

The UF membrane column was split into three segments (front, middle, and rear) using an electric cutter. Following the UF membrane column opening, a yellow filter cake layer adhered to certain areas of the membrane sample surface (Figure 3). Membrane filaments were used for cleaning and surface fouling characterization and analysis. The mid-membrane filament samples were cut into equal-length sections for testing.

#### 2.3.2. Membrane Porosity

Membrane volumetric porosity ε (%) is defined as the pore volume divided by the total volume of the membrane. Membrane porosity significantly influences the adsorption and transfer processes, as well as affecting the contact area between foulants and cleaning agents. This makes it a crucial factor in evaluating the filtration efficiency, flux performance, and separation capability of the membrane [30]. The quality of foulant removal is indirectly characterized by the change in weight of the fouled membrane before and after cleaning [31]. To determine the effect of fouling removal by weight change, membrane volumetric porosity can be determined by the weight method, measuring the weight of the liquid (pure water) contained in the membrane pores. The volumetric porosity of the membrane is calculated by Equation (1).

(1) ε=Ww-WdpwWw-Wdpw+Wdpρ

where W_W_ is the weight of the wet membrane, W_d_ is the weight of the dry membrane, p_w_ is the density of water, which is 1 g/cm^3^, and p_p_ is the density of the membrane, which is 0.96 g/cm^3^.

#### 2.3.3. Organic Foulant Analysis

Total organic carbon (TOC) analyzer (TOC-L CPH, SHIMA-DZU, Kyoto, Japan) was used to analyze the TOC of the UF eluates [32]. Three-dimensional fluorescence spectrometry was measured by the fluorescence excitation–emission matrix (EEM) (F-7000, Hitachi, Tokyo, Japan) [33,34]. Organic species in the eluate can be characterized qualitatively by measuring the fluorescence intensity, which is usually categorized into five fluorescence regions (Table 3) [35]. An attenuated total reflectance Fourier-transform infrared spectroscope (ATR-FTIP) (Magna-IR 750, Nicolet, Green Bay, WI, USA) was used to analyze the functional groups on the membrane surface.

#### 2.3.4. Inorganic Foulant Analysis

Inductively coupled plasma (ICP) (ICP-OESOPTIMA-2000, PerkinElmer, Waltham, MA, USA) was used for the determination of inorganics in the membrane eluent [36]. A scanning electron microscope (SEM) (JSM-7800F, JEOL, Tokyo, Japan) was used to examine the surface morphology. The elemental changes in the membrane surface were analyzed using an energy-dispersive spectrometer (EDS) (Spectra 300, Thermo Fisher, Waltham, MA, USA).

## 3. Results and Discussion

### 3.1. Membrane Foulant Identification and Comparison of Different Cleaning Effects

#### 3.1.1. Analysis of Foulants on the Membrane Surface

**Figure 4:**
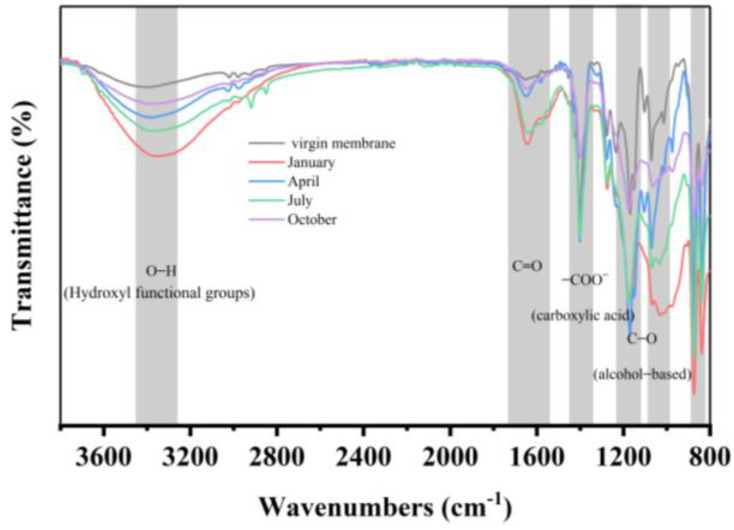
Infrared spectrum of the fouled membranes in different seasons.

The changes in the membrane surface functional groups of the fouled membrane filaments in different seasons are shown in Figure 4. The absorption peak near 840 cm^−1^ was attributed to CH out-of-plane deformation and CH_2_ rocking [37], and the absorption peak near 895 cm^−1^ was characteristic of CF_2_ symmetric stretching [38]; these were the characteristic peaks for PVDF. There was no seasonal variation for the fouled membranes. The absorption peaks at 1075 cm^−1^ and 1181 cm^−1^ were attributed to the C–O stretching of alcohols originating from polysaccharides [39], and the absorption peak at 1430 cm^−1^ was attributed to the stretching vibrations of –COO^−^ associated with carboxylic acid [40]. Meanwhile, the specific peaks related to HA appeared at 1640 cm^−1^, owing to C=O stretching [41]. The broad region of the absorption peak between 3288 cm^−1^ and 3450 cm^−1^ in the fouled membrane and the virgin membrane was caused by the stretching oscillation of hydroxyl functional groups (O–H) [42]. At the same time, HA also responds to this position [43,44]. The results show that the fouled membrane mainly contains HA.

**Figure 5:**
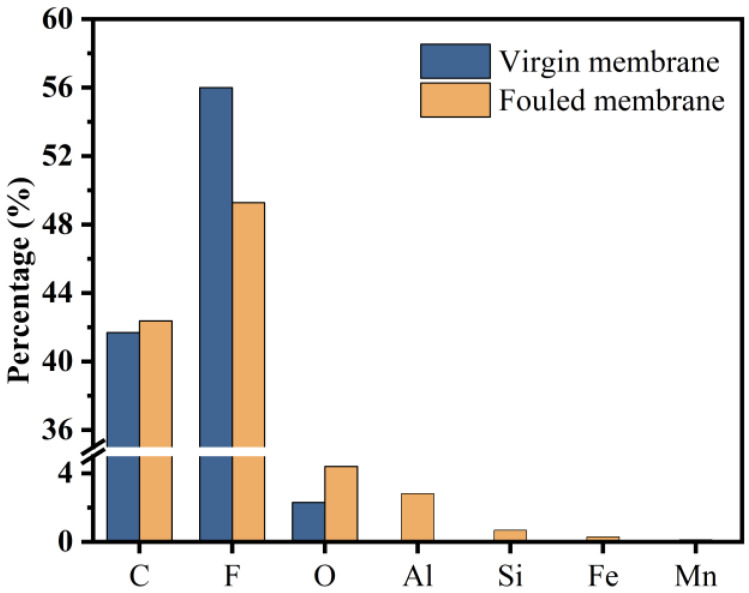
Comparison of the relative mass of elements between the virgin membrane and the fouled membranes (summer—July).

The results of EDS are shown in Figure 5. For the fouled membranes taken from summer, several oxides such as Al, Si, Fe, and Mn were detected with Al and Si as the main foulants. Therefore, it can be preliminarily determined that the type of membrane fouling was mainly complex fouling.

Al fouling is mainly due to the use of poly Al chloride in the coagulation process of this DWTP, which enriches the Al content in the membrane tank considerably. Since the content of Si compounds in raw water is maintained at about 10 mg/L, it is speculated that the cause of Si fouling may be that the Si in raw water adheres to the surface of the membrane, which forms after long-term filtration and accumulation [45].

#### 3.1.2. Effects of Different Chemical Cleaning Types

According to the membrane surface foulant analysis, the presence of inorganics and organics constitutes complex fouling. To further verify the foulants, sodium citrate, NaClO, the NaClO + NaOH combination, and ultrasonic cleaning were used to clean the fouled membrane, and the composition of the eluates was analyzed.

**Figure 6:**
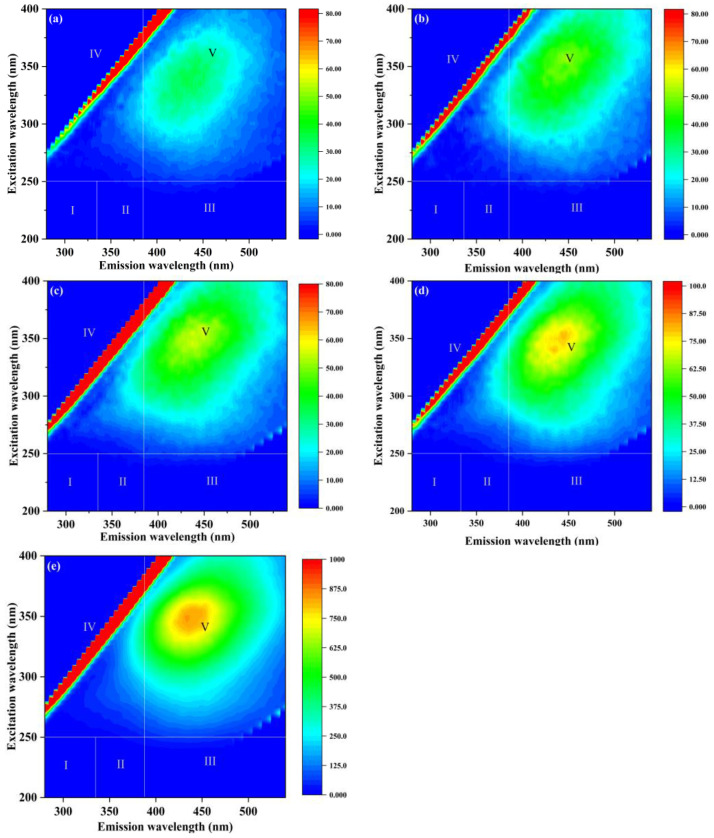
EEM spectra: (**a**) raw water, (**b**) ultrasonic eluate, (**c**) sodium citrate eluate, (**d**) NaClO eluate, and (**e**) NaClO + NaOH eluate.

**Figure 7:**
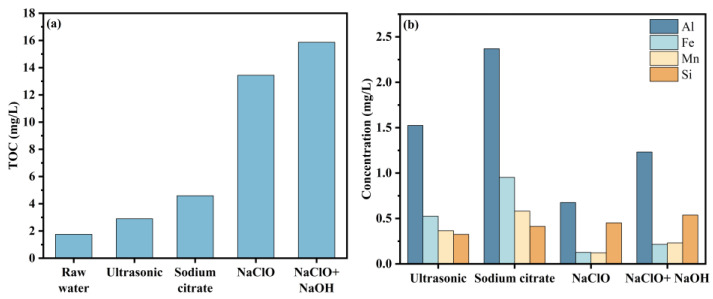
Foulant in the eluate: (**a**) TOC and (**b**) inorganic element (the TOC of the sodium citrate eluate is obtained by subtracting the TOC of the control group).

The EEM spectra show that the organics in the eluant are mainly HA (Figure 6) [46]. Figure 6 also indicates that chemical cleaning is more effective than physical cleaning to remove HA fouling. The characteristic peaks were significantly different in different eluates. The combination of NaClO + NaOH has a better effect on the elution of organics than other cleaning. Figure 7a shows that the removal efficiency of NaClO and NaClO + NaOH on HA was significantly better than that of sodium citrate and ultrasonic cleaning.

Sodium citrate cleaning performed better than NaClO, NaClO + NaOH, and ultrasonic cleaning on the removal of metal elements (Al, Fe, Mn) (Figure 7b). However, the four methods are similar for the removal of Si.

It can be determined that the foulants are inorganics, mainly composed of Al and Si, and organics, mainly composed of HA.

### 3.2. Volume Porosity Changes in Chemically Cleaned Membrane

**Figure 8:**
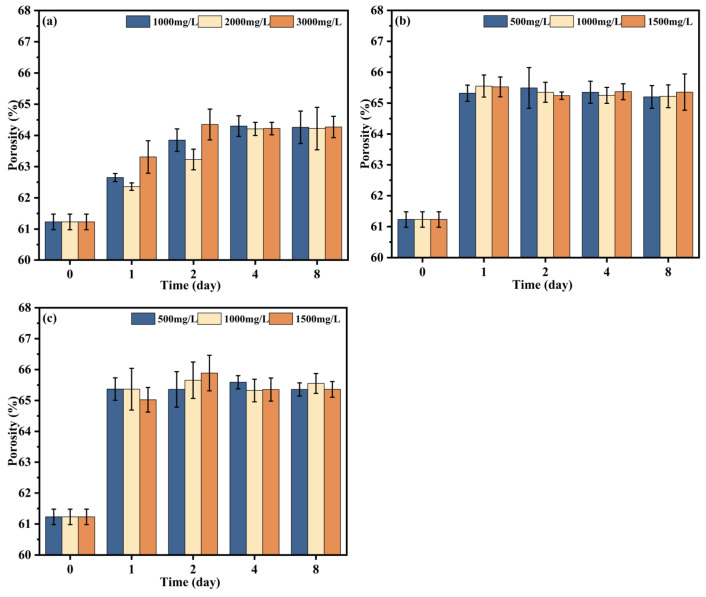
Volume porosity of fouled membranes cleaned with different chemicals: (**a**) sodium citrate, (**b**) NaClO, and (**c**) NaClO + NaOH.

Membrane porosity has an impact on the membrane flux performance [47]. Figure 8 shows the volume porosity of the fouled membrane cleaned with different chemical reagents, namely, sodium citrate, NaClO, and NaClO combined with 250 mg/L NaOH, at different times. From the porosity change in the membrane after sodium citrate cleaning, it can be found that the overall porosity is stable and no longer rises after 2 days of cleaning, indicating that the membrane pore blockage has been removed at this time. On the other hand, the porosity of NaClO alone and that of the combined cleaning are both stable and no longer increases after 1 day of cleaning, and the overall stability value of porosity is higher than that of sodium citrate cleaning. It is speculated that the main substance in the fouled membrane is HA fouling, so NaClO has a better removal effect [48]. According to the change in porosity at different concentrations and times, the overall chemical concentration has less effect on porosity than cleaning time, which is consistent with the conclusion that the cleaning time has more effect than the concentration on fouled membranes in some studies [49]. Routine cleaning procedures and chemical expenses at DWTPs should not be underestimated. The concentration of sodium citrate, NaClO, and NaOH in the follow-up chemical cleaning experiment were 1000 mg/L, 500 mg/L, and 250 mg/L.

### 3.3. Impact of Various Chemicals on Organic and Inorganic Removal and Membrane Morphology

#### 3.3.1. Effectiveness of Chemical Cleaning on the Removal of Organics

(1) Component of chemical elutriate

**Figure 9:**
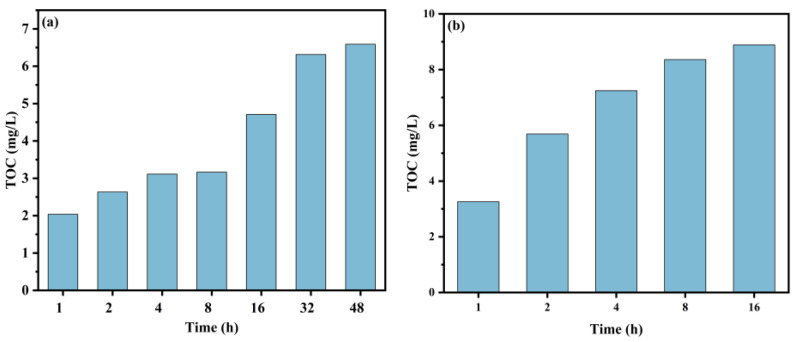
Changes in TOC of eluates with cleaning time: (**a**) NaClO and (**b**) NaClO + NaOH.

Alkaline cleaning is mainly used to remove organics from the membrane surface, and the composition of the eluate represents the changes in its organics. Figure 9a shows that the TOC in the eluates of the membrane does not increase significantly after 32 h of cleaning. Figure 9b shows that the TOC of the eluates does not change significantly after the NaClO + NaOH cleaning time reaches 8 h. Compared with NaClO cleaning alone, the cleaning time is greatly shortened.

#### 3.3.2. Effectiveness of Chemical Cleaning on the Removal of Inorganics

(1) Component of chemical elutriate

**Figure 10:**
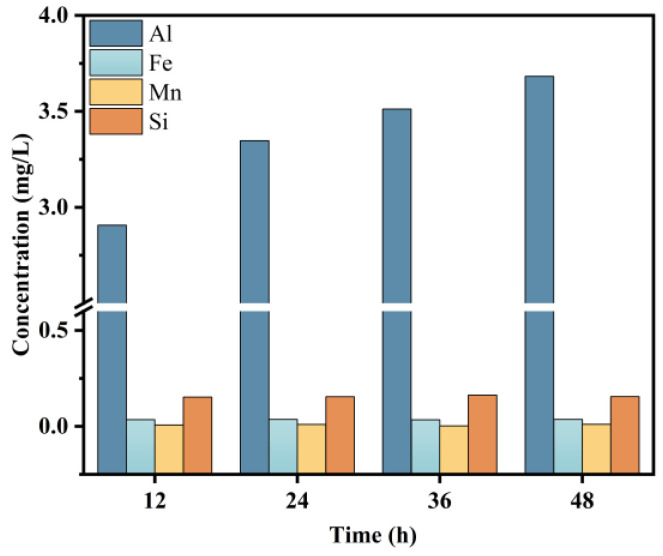
Metal ion concentration in eluates over time.

Acid cleanings are mainly used to remove inorganics, particularly metal oxides, and the removal mechanism comprises hydrolyzing the metal oxides on the fouled membrane at a low pH [50]. Al is the main element in the fouled components of the inorganic membrane, accounting for more than 95% of the content of inorganic elements in the foulant. After 24 h of sodium citrate cleaning, the Al content in the eluate does not significantly increase and tends to be stable at 48 h (Figure 10). It is preliminarily speculated that the inorganics (mainly Al) have been completely cleaned and shed [51,52].

(2) Analysis of membrane surface elements after cleaning

**Figure 11:**
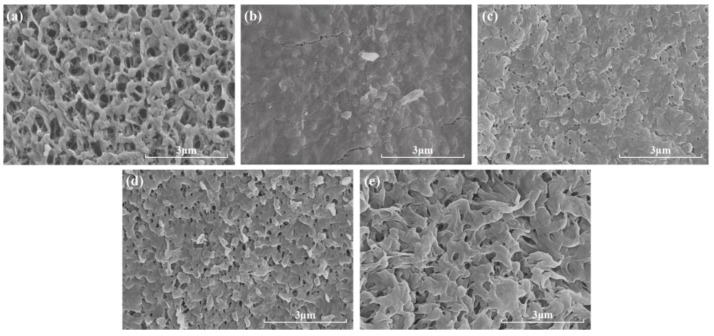
SEM images of membrane before and after chemical cleaning: (**a**) virgin membrane, (**b**) fouled membrane, (**c**) sodium citrate, (**d**) NaClO, and (**e**) NaClO + NaOH.

Understanding the morphological properties of the membrane is important for further understanding its fouling behavior and retention performance. Figure 11 presents SEM images of the membranes before and after chemical cleaning. Before cleaning, the surface of the fouled membrane was covered with a large amount of foulant, and the distribution of membrane pores could not be observed (Figure 11b). Comparing the cleaning effect of different chemicals, after cleaning with NaClO + NaOH, the membrane surface exhibited the least fouling, the membrane pores were exposed, and the surface texture was distributed.

The results obtained via the EDS are shown in Table 4. Sodium citrate has a good effect on the removal of inorganic metal foulants, such as Al, Fe, and Mn, but it has a poor effect on the removal of Si substances. It is worth noting that the content of C and O also declined. It is speculated that this declining content is organic fouling, which shows that sodium citrate could remove part of organics after a long period of soaking and cleaning. This is because the chelation of sodium citrate loosens the dense fouling layer, and then, the organics can be removed together with inorganics [53,54,55].

The relative mass of the main foulants before and after the NaClO cleaning of the fouled membrane did not significantly decrease, indicating that NaClO has a limited effect on the removal of inorganic elements and oxides from the surface of the membrane. Compared with NaClO cleaning alone, the main inorganic fouling elements, such as Al Si, Fe, and Mn, are reduced to varying degrees after cleaning with NaClO + NaOH. The removal effect of Al and Si is significantly better than that of NaClO cleaning alone, in terms of the mass distribution of elements on the surface of the membrane. The main reason is that Al and Si easily form soluble salts under strong alkalinity and, thus, leave the membrane surface [56]. Because of the addition of NaOH, the eluate is always in a strong alkaline state, so that the Al and Si elements in the membrane foulant are easily converted into soluble salts and, thus, detach from the surface of the membrane [57]. NaOH is more likely to reach the organic fouled layer, improving the cleaning efficiency.

### 3.4. Cleaning Verification

#### 3.4.1. Full-Scale Cleaning Tests

**Figure 12:**
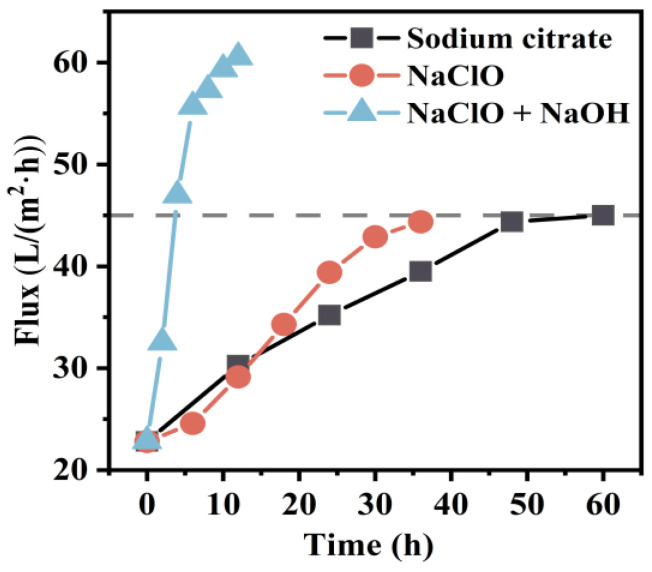
Variation in membrane flux with cleaning time in full-scale tests.

The efficacy of cleaning chemicals was analyzed through bench-scale tests, and based on these findings, the recovery of membrane flux was further evaluated in full-scale tests to assess the impact of chemical cleaning on the fouled membranes. The cleaning solution was drained every 12 h cleaning, and the membrane tank was rinsed to eliminate any residue, ensuring that no cleaning solution remained. Water production was maintained at a constant pressure of 30 kPa, and the corresponding stable flux changes were recorded. The overall membrane flux increased gradually as sodium citrate cleaning progressed, but the rate of flux recovery slowed down over time. After 48 h of cleaning, there was no significant further increase in flux, indicating that most inorganic foulants had been effectively removed (Figure 12). For NaClO cleaning, membrane flux ceased to recover beyond 32 h, suggesting that the majority of HA had been eliminated from the membrane surface. For the combined NaClO + NaOH cleaning, the overall cleaning time was shortened significantly to 8 h.

#### 3.4.2. Engineering Case

**Figure 13:**
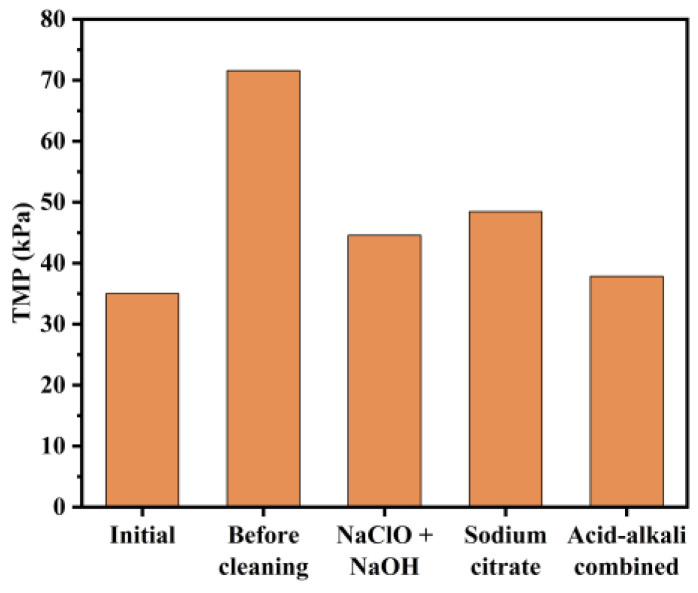
Changes in TMP before and after membrane chemical cleaning with different cleaning reagents in DWTP.

The analysis of bench-scale tests, eluate composition, and flux verification from the full-scale tests indicate that the most effective cleaning strategy is to first treat the fouled membrane with NaClO + NaOH, followed by sodium citrate. In the engineering tests conducted at the DWTP, the selected cleaning protocol involved using 500 mg/L NaClO + 250 mg/L NaOH, followed by 1000 mg/L sodium citrate. As shown in Figure 13, TMP recovery after sodium citrate cleaning reached 48.45 kPa, compared to 44.56 kPa after NaClO + NaOH cleaning, demonstrating that the combined cleaning approach was more effective than sodium citrate alone. Moreover, TMP recovery after acid–alkaline cleaning was as high as 37.82 kPa, indicating that the combination cleaning method had superior performance. The results of the acid–alkali combined cleaning in the engineering tests were consistent with those observed in full-scale tests, confirming its effectiveness.

## 4. Conclusions

In this work, membrane-fouling control strategies for DWTPs were evaluated through chemical cleaning tests conducted at bench-scale, full-scale, and engineering case levels. The key findings are as follows.

Analyzing the fouling membrane after chemical cleaning and its eluate indicates that proteins, polysaccharides, and HA contributed to membrane fouling, with HA being the predominant organic foulant. The main inorganic foulants were identified as Al and Si.

Bench-scale tests reveal that sodium citrate was more effective in removing inorganic fouling, while NaClO was more efficient in eliminating organic fouling, particularly HA. Additionally, the combined NaClO + NaOH cleaning demonstrate superior performance over NaClO alone, especially in removing Si-based fouling.

Full-scale tests show that the acid–alkali combined cleaning approach achieved the highest flux recovery, increasing the membrane flux from 22.8 L/(m^2^·h) to 66.75 L/(m^2^·h) at a constant pressure of 30 kPa. Furthermore, the NaClO + NaOH cleaning method significantly reduced the required alkali washing time compared to NaClO alone. The results from the engineering tests are consistent with the full-scale tests, confirming the efficacy of the combined acid–alkali cleaning approach.

The results from the engineering tests are consistent with the full-scale tests, and the combined acid–alkali cleaning could obtain a higher recovery rate while shortening the cleaning time. Therefore, the combined acid–alkali of NaClO (500 mg/L) + NaOH (250 mg/L) cleaning approach followed by sodium citrate (1000 mg/L) is adopted in the actual application, which can achieve a better membrane cleaning effect.

## Figures and Tables

**Table 1:** Seasonal main water quality indicators of raw water from DWPT (202303-202402).

Parameters	Spring March–May	Summer June–August	Autumn September–November	Winter December–February
pH	7.08–7.4	6.88–7.19	6.96–7.10	7.32–7.50
Temperature (°C)	12.9–16.3	20.1–26.0	19.6–25.9	11.1–14.7
Turbidity (NTU)	1.17–2.00	2.25–28.6	3.42–5.57	1.58–3.66
COD_Mn_ (mg/L)	1.4–1.5	1.6–2.8	1.5–2.3	1.7–2.5
Fe (mg/L)	0.04–0.06	0.06–0.52	0.11–0.19	0.08–0.17
Mn (mg/L)	0.04–0.09	0.05–0.38	0.12–0.22	0.06–0.22
Alkalinity (mg/L)	22.1–23.5	18.4–26.4	22.5–25.6	28.0–29.1
Hardness (mg/L)	25–26	21–28	26–28	31–32

**Table 2:** Main performance parameters of membrane materials.

Membrane Materials	Modified Material	Pore Size/μm	Inside and Outside Diameter/mm	Temperature Range/°C	Maximum Tolerated Concentration (NaClO)/mg/L
PVDF	PVP	0.03	0.7/1.2	5~40	5000

**Table 3:** Fluorescent area and characteristic substances.

Area	Fluorescent Area	Characteristic Substances
Ⅰ	E_X_200–250 nm E_M_280–330 nm	Tyrosine
Ⅱ	E_X_200–250 nm E_M_330–380 nm	Aromatic protein
Ⅲ	E_X_200–250 nm E_M_380–500 nm	Fulvic acids
Ⅳ	E_X_250–320 nm E_M_280–380 nm	Tyrosine
Ⅴ	E_X_280–350 nm E_M_380–500 nm	HA

**Table 4:** Changes in the mass distribution of elements on the surface of membranes before and after cleaning with different chemicals.

Chemicals	C (%)	F (%)	O (%)	Al (%)	Si (%)	Fe (%)	Mn (%)
Before cleaning	42.38	49.29	4.41	2.82	0.68	0.30	0.12
Sodium citrate	40.32	58.55	1.25	0.26	0.62	0	0
NaClO	43.20	50.80	3.89	1.18	0.53	0.30	0.10
NaClO + NaOH	41.59	55.56	2.31	0.43	0.05	0.06	0.02

## Data Availability

Data are contained within the article.

## References

[1] Malkoske T.A., Bérubé P.R., Andrews R.C. (2020). Coagulation/flocculation prior to low pressure membranes in drinking water treatment: A review. Environ. Sci. Water Res. Technol..

[2] Wang Y., Ma B., Ulbricht M., Dong Y., Zhao X. (2022). Progress in alumina ceramic membranes for water purification: Status and prospects. Water Res..

[3] Kumar R., Ismail A.F. (2015). Fouling control on microfiltration/ultrafiltration membranes: Effects of morphology, hydrophilicity, and charge. J. Appl. Polym. Sci..

[4] Qi J., Ma B., Miao S., Liu R., Hu C., Qu J. (2021). Pre-oxidation enhanced cyanobacteria removal in drinking water treatment: A review. J. Environ. Sci..

[5] Li K., Li S., Huang T., Dong C., Li J., Zhao B., Zhang S. (2019). Chemical Cleaning of Ultrafiltration Membrane Fouled by Humic Substances: Comparison between Hydrogen Peroxide and sodium Hypochlorite. Int. J. Environ. Res. Public. Health.

[6] Gao W., Liang H., Ma J., Dong C., Li J., Zhao B., Zhang S. (2011). Membrane fouling control in ultrafiltration technology for drinking water production: A review. Desalination.

[7] Wang Q., Wang Z., Wu Z. (2012). Effects of solvent compositions on physicochemical properties and anti-fouling ability of PVDF microfiltration membranes for wastewater treatment. Desalination.

[8] Luo H., Wang Z. (2022). A new ultrasonic cleaning model for predicting the flux recovery of the UF membrane fouled with humic acid. J. Environ. Chem. Eng..

[9] Gan X., Lin T., Jiang F., Zhang X. (2021). Impacts on characteristics and effluent safety of PVDF ultrafiltration membranes aged by different chemical cleaning types. J. Membr. Sci..

[10] Fu W., Pei T., Mao Y., Li G., Zhao Y., Chen L. (2019). Highly hydrophilic poly(vinylidene fluoride) ultrafiltration membranes modified by poly (N-acryloyl glycinamide) hydrogel based on multi-hydrogen bond self-assembly for reducing protein fouling. J. Membr. Sci..

[11] Jiang H., Zhao Q., Wang P., Chen M., Wang Z., Ma J. (2021). Inhibition of algae-induced membrane fouling by in-situ formed hydrophilic micropillars on ultrafiltration membrane surface. J. Membr. Sci..

[12] Yusuf A., Sodiq A., Giwa A., Eke J., Pikuda O., De Luca G., Di Salvo J.L., Chakraborty S. (2020). A review of emerging trends in membrane science and technology for sustainable water treatment. J. Clean. Prod..

[13] Zhang J., Li G., Yuan X., Li P., Yu Y., Yang W., Zhao S. (2023). Reduction of ultrafiltration membrane fouling by the pretreatment removal of emerging pollutants: A review. Membranes.

[14] Xu C., Liu X., Xie B., Yao C., Hu W., Li Y., Li X. (2016). Preparation of PES ultrafiltration membranes with natural amino acids based zwitterionic antifouling surfaces. Appl. Surf. Sci..

[15] Yue X., Koh Y.K.K., Ng H.Y. (2015). Effects of dissolved organic matters (DOMs) on membrane fouling in anaerobic ceramic membrane bioreactors (AnCMBRs) treating domestic wastewater. Water Res..

[16] Ma B., Ding Y., Wang B., Qi Z., Bai Y., Liu R., Liu H., Qu J. (2020). Influence of sedimentation with pre-coagulation on ultrafiltration membrane fouling performance. Sci. Total Environ..

[17] Chen H., Huang M., Liu Y., Meng L., Ma M. (2020). Functionalized electrospun nanofiber membranes for water treatment: A review. Sci. Total Environ..

[18] Kilmer N.T., Huss R.L., George C.C., Stennett E.M. (2021). The influence of ion identity and ionic strength on membrane biofouling of a binary protein solution. Sep. Purif. Technol..

[19] Santoro S., Tufa R.A., Avci A.H., Fontananova E., Di Profio G., Curcio E. (2021). Fouling propensity in reverse electrodialysis operated with hypersaline brine. Energy.

[20] Petrosino F., De Luca G., Curcio S., Wickramasinghe S.R., Chakraborty S. (2023). Micro-CFD modelling of ultrafiltration bio-fouling. Sep. Sci. Technol..

[21] Tanudjaja H.J., Anantharaman A., Ng A.Q.Q., Ma Y., Tanis-Kanbur M.B., Zydney A.L., Chew J.W. (2022). A review of membrane fouling by proteins in ultrafiltration and microfiltration. J. Water Process Eng..

[22] Peters C.D., Rantissi T., Gitis V., Hankins N.P. (2021). Retention of natural organic matter by ultrafiltration and the mitigation of membrane fouling through pre-treatment, membrane enhancement, and cleaning-A review. J. Water Process Eng..

[23] Gruskevica K., Mezule L. (2021). Cleaning methods for ceramic ultrafiltration membranes affected by organic fouling. Membranes.

[24] Xiao T., Zhu Z., Li L., Shi J., Li Z., Zuo X. (2023). Membrane fouling and cleaning strategies in microfiltration/ultrafiltration and dynamic membrane. Sep. Purif. Technol..

[25] Liang H., Gong W., Chen J., Li G. (2008). Cleaning of fouled ultrafiltration (UF) membrane by algae during reservoir water treatment. Desalination.

[26] Ding W., Chen M., Zhou M., Zhong Z., Cui Z., Xing W. (2020). Fouling behavior of poly (vinylidene fluoride) (PVDF) ultrafiltration membrane by polyvinyl alcohol (PVA) and chemical cleaning method. Chin. J. Chem. Eng..

[27] Wang L., Wang Q., Li Y., Lin H. (2013). Ultrasound-assisted chemical cleaning of polyvinylidene fluoride membrane fouled by lactic acid fermentation broth. Desalination.

[28] Brepols C., Drensla K., Janot A., Trimborn M., Engelhardt N. (2008). Strategies for chemical cleaning in large scale membrane bioreactors. Water Sci. Technol..

[29] Wang Q., Wang Z., Sun Q., Yang Z., Wang Z., Wu L. (2022). Anti-fouling performance investigation of micron-submicron hierarchical structure PVDF membranes in water-in-oil emulsion separation. J. Environ. Chem. Eng..

[30] Zhang Z., Gao J., Zhang W., Ren Z. (2006). Experimental study of the effect of membrane porosity on membrane absorption process. Sep. Sci. Technol..

[31] Xiao M., Yang F., Im S., Dlamini D.S., Jassby D., Mahendra S., Honda R., Hoek E.M. (2022). Characterizing surface porosity of porous membranes via contact angle measurements. J. Membr. Sci. Lett..

[32] Bisutti I., Hilke I., Raessler M. (2004). Determination of total organic carbon–an overview of current methods. TrAC Trends Anal. Chem..

[33] Mahmoud A.A.A., Elkatatny S., Mahmoud M., Abouelresh M., Abdulraheem A., Ali A. (2017). Determination of the total organic carbon (TOC) based on conventional well logs using artificial neural network. Int. J. Coal Geol..

[34] Nakaya Y., Nakashima S., Moriizumi M., Oguchi M., Kashiwagi S., Naka N. (2020). Three dimensional excitation-emission matrix fluorescence spectroscopy of typical Japanese soil powders. Spectrochim. Acta Part. A Mol. Biomol. Spectro..

[35] Ma K., Shen H., Zhou T., Xin H., Wu F., Zhang G. (2023). Water quality characteristics and evaluation of Qilian Mountain National Park section in Heihe River Basin based on water quality indices and 3D fluorescence technology. Environ. Geochem. Health.

[36] Hong A., Tang Q., Khan A.U., Miao M., Xu Z., Dang F., Liu Q., Wang Y., Lin D., Filser J. (2021). Identification and speciation of nanoscale silver in complex solid matrices by sequential extraction coupled with inductively coupled plasma optical emission spectrometry. Anal. Chem..

[37] Elashmawi I.S. (2008). Effect of LiCl filler on the structure and morphology of PVDF films. Mater. Chem. Phys..

[38] Chen M., Ding W., Zhou M., Zhang H., Ge C., Cui Z., Xing W. (2021). Fouling mechanism of PVDF ultrafiltration membrane for secondary effluent treatment from paper mills. Chem. Eng. Res. Des..

[39] Fan C., Yan J., Liu H., Xie Y., Liu H. (2024). Performance and membrane fouling characteristics of a drinking water multistage NF system based on membrane autopsy from a full-scale system. J. Water Process Eng..

[40] Cheng X., Li P., Zhu X., Luo C., Zheng L., Hou C., Wu D., Liang H. (2021). Role of different dimensional carbon nanoparticles in catalytic oxidation of organic pollutants and alleviating membrane fouling during ultrafiltration of surface water. Sep. Purif. Technol..

[41] Zhang M., Hong H., Lin H., Shen L., Yu H., Ma G., Chen J., Liao B.-Q. (2018). Mechanistic insights into alginate fouling caused by calcium ions based on terahertz time-domain spectra analyses and DFT calculations. Water Res..

[42] Lee Y.G., Shin J., Kim S.J., Cho K.H., Westerhoff P., Rho H., Chon K. (2023). An autopsy study of hollow fiber and multibore ultrafiltration membranes from a pilot-scale ultra high-recovery filtration system for surface water treatment. Sci. Total Environ..

[43] Fan L., Harris J.L., Roddick F.A., Booker N.A. (2001). Influence of the characteristics of natural organic matter on the fouling of microfiltration membranes. Water Res..

[44] Howe K.J., Ishida K.P., Clark M.M. (2002). Use of ATR/FTIR spectrometry to study fouling of microfiltration membranes by natural waters. Desalination.

[45] Lian J., Cheng X., Zhu X., Luo X., Xu J., Tan F., Wu D., Liang H. (2023). Mutual activation between ferrate and calcium sulfite for surface water pre-treatment and ultrafiltration membrane fouling control. Sci. Total Environ..

[46] Gryta M. (2007). Influence of polypropylene membrane surface porosity on the performance of membrane distillation process. J. Membr. Sci..

[47] Hosseini P.K., Liu L., Hosseini M.K., Bhattacharyya A., Miao J., Wang F. (2023). Treatment of a synthetic decanted oily seawater in a pilot-scale hollow fiber membrane filtration process: Experimental investigation. J. Hazard. Mater..

[48] Yan M., Guo K., Gao Y., Yue Q., Gao B. (2023). Insights into the control mechanism of different coagulation pretreatment on ultrafiltration membrane fouling for oily wastewater treatment. Sep. Purif. Technol..

[49] Zueva O.S., Khair T., Kazantseva M.A., Latypova L., Zuev Y.F. (2023). Ions-Induced Alginate Gelation According to Elemental Analysis and a Combinatorial Approach. Int. J. Mol. Sci..

[50] Xue W., Jian M., Lin T., Ma B., Wu R., Li X. (2020). A novel strategy to alleviate ultrafiltration membrane fouling by rotating membrane module. Chemosphere.

[51] Zhu C., Wang H., Ma H., Yang Y., Li F. (2020). Tanning process promotes abiotic humification: Separation and characterization of humic acid-like polymers complex. Environ. Sci. Pollut. Res..

[52] Du P., Li X., Yang Y., Zhou Z., Fan X. (2020). Dual coagulation with floc breakage to alleviate ultrafiltration membrane fouling caused by algae organic matter. Desalination.

[53] Jiménez-González M.A., Álvarez A.M., Carral P., Almendros G. (2019). Chemometric assessment of soil organic matter storage and quality from humic acid infrared spectra. Sci. Total Environ..

[54] Wan Y., Xie P., Wang Z., Wang J., Ding J., Dewil R., Van der Bruggen B. (2021). Application of UV/chlorine pretreatment for controlling ultrafiltration (UF) membrane fouling caused by different natural organic fractions. Chemosphere.

[55] Liu B., Yin J., Wu J., Cheng X., Yang K., Li G., Shi Z. (2022). Effect of UV/ClO2 pretreatment on controlling ultrafiltration membrane fouling of different natural organic matter (NOM) fractions. J. Water Process Eng..

[56] Holbrook R.D., DeRose P.C., Leigh S.D., Rukhin A.L., Heckert N.A. (2006). Excitation–emission matrix fluorescence spectroscopy for natural organic matter characterization: A quantitative evaluation of calibration and spectral correction procedures. Appl. Spectrosc..

[57] Zondervan E., Roffel B. (2007). Evaluation of different cleaning agents used for cleaning ultra filtration membranes fouled by surface water. J. Membr. Sci..

